# The Association Between the Sedative Loads and Clinical Severity Indicators in the First-Onset Major Depressive Disorder

**DOI:** 10.3389/fpsyt.2019.00129

**Published:** 2019-03-18

**Authors:** Yen-Chin Wang, Hai-Ti Lin, Mong-Liang Lu, Ming-Chyi Huang, Chun-Hsin Chen, Tzu-Hua Wu, Sabrina Wang, Wei-Chung Mao, Po-Hsiu Kuo, Hsi-Chung Chen

**Affiliations:** ^1^Department of Psychiatry, National Taiwan University Hospital, Taipei, Taiwan; ^2^Department of Psychiatry, Wan-Fang Hospital & School of Medicine, College of Medicine, Taipei Medical University, Taipei, Taiwan; ^3^Department of Psychiatry, Taipei City Hospital, Songde Branch, Taipei, Taiwan; ^4^Department of Clinical Pharmacy, School of Pharmacy, College of Pharmacy, Taipei Medical University, Taipei, Taiwan; ^5^School of Medicine, Institute of Anatomy and Cell Biology, National Yang-Ming University, Taipei, Taiwan; ^6^Department of Psychiatry, Cheng-Hsin General Hospital & School of Medicine, National Defense Medical Center, Taipei, Taiwan; ^7^Graduate Institute of Epidemiology and Preventive Medicine, College of Public Health, National Taiwan University, Taipei, Taiwan; ^8^Center of Sleep Disorders, National Taiwan University Hospital, Taipei, Taiwan

**Keywords:** major depressive disorder, sedatives, psychotropic load, pharmacological dissection, clinical specifier

## Abstract

**Background:** High sedative use in a major depressive episode may imply specific clinical features. This study aims to examine the correlation between sedative use and clinical severity indicators in the initial treatment phase of first-onset major depressive disorder.

**Methods:** A study cohort in the first episode of major depressive disorder was used to conduct pharmacological dissection. All participants had at least a 2-year follow-up period with a complete treatment record. The defined daily dose of antidepressants and augmentation agents were calculated as the antidepressant load and augmentation load, respectively. Sedative use, which was calculated as the equivalent dosage of lorazepam, were defined as the sedative load. These psychotropic loads were measured monthly and the averaged psychotropic loads for each day were obtained.

**Results:** A total of 106 individuals (75.5% female) were included. The mean duration of disease course in participants was 5.5 ± 3.5 years. In the multiple regression analysis, after controlling for other classes of psychotropics and comorbid anxiety disorders, the sedative load independently correlated with higher number of antidepressants used, higher number of antidepressant used with an adequate dose and duration, more psychiatric emergency and outpatient visits within 2 years of disease onset.

**Conclusion:** High loading of sedatives correlated with several indicators of clinical severity in major depressive disorder. The sedative load may be used as a specifier to identify subgroups in patients with major depressive disorder.

## Introduction

The high heterogeneity of major depressive disorder (MDD) imposes complexities on sub-grouping treatment response (1), impacts the study of endophenotype and genotype indirectly (2–4), and further compromises delivery of specific antidepressant treatment. Although several clinical specifiers have been identified as subgroups of MDD, such as melancholic, atypical, and seasonal features, their clinical utility is of limited use due to the theoretical and phenomenological nature of these specifiers (5). Therefore, recognizing non-descriptive specifiers of MDD and linking them with established neurochemical mechanisms would help dissect the pathomechanisms that underlie the causal heterogeneity of MDD (6).

MDD usually co-occurs with anxiety symptoms. In previous studies, more than half of patients with MDD have a comorbid anxiety disorder (7, 8). Patients with MDD and concurrent anxiety symptoms have poorer treatment responses (9–11), longer disease course (11–14), and higher suicidality (13, 15). In parallel, sleep disturbance is also a commonly co-occurring symptom of MDD (16). About 80% patients with MDD have concurrent sleep disturbance. Even during remission, almost 50% of patients with MDD suffer from sleep problems (17). Persistent insomnia has also been shown to predict subsequent relapses of MDD in young adults (18). The co-occurrence of anxiety symptoms and sleep disturbance with MDD are of particular clinical importance.

Many patients receive sedatives to relieve anxiety symptoms and insomnia while also receiving antidepressant treatment for MDD. According to current practice guidelines, the mainstay treatment of co-occurring anxiety and insomnia that co-occur with MDD should be primarily antidepressants; sedatives are used only as short-term, adjuvant treatment (19, 20). For example, the guideline from the United Kingdom National Institute for Health and Care Excellence suggests that the duration of adjuvant sedative use should be no more than 2 weeks (19). However, in real-world practice, the empirical data revealed that a significant proportion of patients received a longer duration of sedative use after initial treatment with simultaneous antidepressants and sedatives. In the literature, about 7.6–60% patients of MDD initiated antidepressant therapy with concurrent sedatives, and 12–48% of them received long-term combined treatment (21–27). It appears that antidepressant-based treatment with short-term adjuvant sedatives does not solve the chronic co-occurring anxiety symptoms and insomnia in some patients with MDD. This suggests that the unintended, intensive exposure to sedatives in MDD treatment may specify a subgroup of patients in whom monotherapy with antidepressants fails to correct their underlying patho-etiology. Accordingly, quantifying the pattern of sedative use and examining its validity with external severity indicators might provide a new insight for the phenotypic classification of MDD.

Thus, the present study intended to pharmacologically dissect psychotropics that were used in the main treatment course of first-episode MDD into different classes. By quantifying the pattern of use of all kinds of psychotropics, the present study aimed to examine the independent relationship between sedative use and indicators of clinical severity.

## Materials and Methods

### Participants

The study cohort was established by the Research Collaborating Group for New Insight, Strategy and Evaluation–Treatment-Resistant Depression Program (RECOGNISE-TRD program). Patients, who were aged from 18 to 65 years, with a DSM-IV-TR diagnosis of MDD were recruited during remission (28). All participants were recruited at two medical centers and one psychiatric hospital in Taiwan from October 2010 to April 2016. Because (1) the natural course of a major depressive episode is about 6–13 months (29), (2) about 80% of patients with their first episode of MDD would recover in 1 year, and (3) more than 90% would recover in 2 years (30), a complete medical record covering at least 2 years was required to allow patients to have adequate opportunity to experience all possible treatment modalities that have been suggested by the treatment guidelines. As a result, a high loading of sedatives in the first 2 years of disease course should reflect the inadequate response of mainstay treatments on residual co-occurring anxiety and insomnia symptoms. Thus, participants were eligible only when they had a complete medical record for at least 2 years from the first onset of MDD.

To examine the predictability of psychotropic load in the 2-year period following disease onset, all participants were followed up for an additional period after 2 years of disease onset to collect information of clinical severity indicators. If the patient discontinued psychiatric treatment because of complete remission before the end of follow-up, the researchers contacted them to ensure that neither major depressive episode nor psychiatric treatment during this interval had occurred. Individuals with incomplete medical records or with a comorbid organic brain syndrome, dementia, substance abuse, psychotic disorder, schizoaffective disorder, or bipolar affective disorder were excluded. Finally, a total of 106 individuals were included. This study was approved by the research ethics committees of each study site. Written informed consent was obtained from each participant.

### Pharmacological Dissection and the Definition of Psychotropics Loads

The psychiatric medications used to treat MDD may be complex but can be classified into antidepressants, augmentations (e.g., anticonvulsants, antipsychotics, lithium, thyroxin, and methylphenidate), and sedatives. To disentangle the specific role of each class of psychotropic, the concept of pharmacological dissection was used (31, 32).

Firstly, the classes and dosage of psychotropic agents that had been prescribed in the first 2 years of treatment were recorded following a chart review. The documented antidepressants included selective serotonin reuptake inhibitors, serotonin-norepinephrine reuptake inhibitors, tricyclic antidepressants, mirtazapine, bupropion, moclobemide, and agomelatine. The augmentation agents included anticonvulsants, antipsychotics, lithium, thyroxin, and methylphenidate. The sedatives included benzodiazepines (BZDs), zaleplon, zolpidem, zopiclone, and eszopiclone. Secondly, the quantity of antidepressants and augmentations was standardized by calculating the defined daily dose (DDD). The DDD was developed in the Anatomical Therapeutic Chemical (ATC) system by the World Health Organization Collaborating Center for Drug Statistics Methodology as the assumed average maintenance dose per day for a drug used for its main indication in adults (33). The cumulative DDD of antidepressants and augmentation agents were calculated and recorded in 4-week intervals. Because the DDD of sedatives could not represent properly the clinical usage of benzodiazepines as anxiolytics or hypnotics (34), this study converted all types of sedatives into equivalent dose of lorazepam, according to the Ashton manual (35). The method of equivalent conversion has been widely used in clinical studies (36–38), clinical practice (39, 40), and epidemiological studies (34). The equivalent doses not provided in the original Ashton manual, including brotizolam, midazolam, oxazolam, and fludiazepam, were determined following the consensus of all researchers involved in the present study. The detailed conversion table is provided as Supplement Table S1. Similarly, the cumulative equivalent dosage of BZDs was calculated using 4-week intervals after the disease onset. In total, 24 data points were retrieved for each class of psychotropic agents in the 2-year period following disease onset. We created three independent variables into regression analysis for each individual, namely the antidepressant load (ADL), augmentation load (AUGL), and sedative load (SL), using the average of daily DDD for antidepressants, augmentations, and cumulative BZD equivalents across 24 months. Because pharmacological dissection for psychotropics was conducted using a chart review and was based on the details of the medication prescription before the end of follow-up, the medical record throughout the follow-up period had to be continuous and complete. If the patient discontinued treatment because of complete remission before the end date of the present study, the psychotropic load was coded as zero. A supplementary figure was provided in the supplementary material (Supplement Figure S1) to illustrate the coding procedure used during the study.

### Identification of Comorbid Anxiety Disorders and Physical Diseases

To partial out the confounding effect of anxiety disorders and medical diseases on the relationship between the SL and clinical severity indicators, the presence of anxiety disorders and number of physical diseases were identified according to the medical records during the initial 2-year period after disease onset. Only DSM-IV-TR anxiety disorders that consecutively appeared more than three times in the medical record were coded as “present” (28). In parallel, medical diseases were numbered according to the list of medical diseases in the Charlson comorbidity index (41).

### Clinical Severity Indicators

To validate the clinical implications for each class of psychotropic load, several clinical indicators were measured to represent specific domains of MDD severity. First, regarding the first 2 years after disease onset, the number of antidepressants that had been prescribed and number of antidepressants that had been used with adequate dosage and adequate duration (ADAD) were counted. The adequate dosage was defined according to the dosage for treating MDD suggested by the Ministry of Health and Welfare, Taiwan (42). An adequate duration was defined as at least 2 weeks (43, 44). The number of used antidepressants reflects how the physicians labored in choosing appropriate antidepressants. In contrast, the number of antidepressants with ADAD indexes the difficulty of treating MDD with antidepressants. In addition, the number of visits to psychiatric outpatient units, acute psychiatric admissions, and uses of psychiatric emergency services were recorded to reflect different domains of disease severity. In the interval from 2 years after disease onset to the end of follow-up, the clinical severity indicators included the number of visits to psychiatric outpatient units and frequency of psychiatric admission. In addition, the severity of depressive symptoms at the end of follow-up was evaluated with the Beck Depression Inventory-II (45). The severity of depression was rescaled according to the cutoffs suggested in Chinese version of Beck Depression Inventory-II: 0 with BDI-II: 0–16 (euthymic), 1 with BDI-II: 17–22 (mild), 2 with BDI-II: 23–30 (moderate), and 3 with BDI-II: 31–63 (severe) (46).

### Statistical Methods

Data were analyzed with the SPSS12.0 statistical package. For the univariate analysis, a Spearman correlation analysis was performed to examine the association between each class of psychotropic loads with various clinical severity indicators. The multicollinearity among psychotropic loads was reviewed and examined by the correlation coefficient rho and the variance inflation factor (VIF). The criteria for signals of collinearity were set at rho ≥ 0.7 and VIF ≥ 5. Except for the severity of depression at the end of follow-up, the clinical severity indicators were all count data. Therefore, Poisson regressions were used to examine the independent relationship of each class of psychotropic load with clinical severity indicators. Regarding with the severity of depression at the end of follow-up, the multiple linear regression was specifically utilized. The statistical significance level was set at *p* < 0.05 in the present study. To examine the influence of multiple tests in the regression analysis, the standard Bonferroni procedure was applied in which a modified significance criterion was used. Accordingly, because there are 5 major independent clinical indicators in our study (i.e., the number of antidepressants used, psychiatric emergency visits, psychiatric admission, psychiatric outpatient visits, and severity of depression), *p* < 0.01 was used as a threshold for the Bonferroni correction.

## Results

Table 1 depicts the basic demographic data and clinical characteristics of the participants. A total of 106 (75.5% female) individuals were included and the mean age was 51.5 ± 13.2 years. The mean age at the initial diagnosis of MDD was 44.5 ± 13.6 years. Regarding comorbidities, 20.8% of the participants had at least one comorbid anxiety disorder. The mean duration of disease of participants before the end of the follow-up was 5.5 ± 3.5 years. The average number of antidepressants use with ADAD was 1.5 ± 0.9. At the end of follow-up, 50% of participants were euthymic and 39.7% had moderate to severe depression. There were no significant differences between gender on ADL (daily average of female: 1.25 ± 0.70, male: 1.23 ± 0.56; *p* = 0.89), AUGL (daily average of female: 0.05 ± 0.10, male: 0.03 ± 0.06; *p* = 0.39), and SL (daily average of female: 2.01 ± 1.95, male: 2.07 ± 2.29; *p* = 0.89).

**Table 1:** Demographic and clinical characteristics of participants (*n* = 106).

	**Mean ± SD**
Age (years)	51.5 ± 13.2
Age of onset (years)	44.5 ± 13.6
Female (n, %)	80 (75.5%)
**PSYCHOTROPICS DISSECTION (MEAN ± SD, RANGE)**	
Comorbid anxiety disorder	22 (20.8%)
Antidepressant load (defined daily dose/day)	1.2 ± 0.7 (range: 0.02–2.9)
Augmentation load (defined daily dose/day)	0.04 ± 0.10 (range: 0.0–0.5)
Sedatives load (equivalent dose/day)	2.0 ± 2.0 (range: 0.0–8.5)
Number of medical diseases	0.7 ± 1.3
**FOLLOW-UP DURATION (YEARS)**	
After disease onset	5.5 ± 3.5
Beyond 2 years of disease onset	3.7 ± 3.5
**CLINICAL FEATURE WITHIN 2-YEAR OF DISEASE ONSET**	
Number of antidepressants use	1.7 ± 0.9
Number of antidepressants use (adequate dosage and duration)	1.5 ± 0.9
Frequencies of psychiatric admission	0.2 ± 0.5
Visits of psychiatric outpatient service	17.3 ± 10.7
Visits of psychiatric emergency service	0.1 ± 0.4
**CLINICAL FEATURE BEYOND 2-YEAR OF DISEASE ONSET**	
Frequencies of psychiatric admission	0.8 ± 0.5
Visits of psychiatric outpatient service	31.1 ± 66.7
**BECK DEPRESSION INVENTORY (*****n*****, %)**	
Euthymic (0–16)	53 (50.0)
Mild (17–22)	11 (10.4)
Moderate (23–30)	20 (18.9)
Severe (31–63)	22 (20.8)

Table 2 summarizes the correlation between the nature of the pharmacological treatment and the use of psychiatric facilities within 2 years of disease onset. The results showed that the ADL, AUGL, and SL positively correlated with each other. All the three categories of psychotropic load positively correlated with the number of antidepressants used and number of antidepressants with ADAD. Furthermore, ADL positively correlated with the visits to psychiatric outpatient units and emergency service use. The AUGL significantly correlated with the number of psychiatric admissions and visits to outpatient units. The SL significantly correlated with the visits to psychiatric outpatient units and emergency service use. The number of antidepressants used with ADAD correlated with all the other clinical severity indexes, but not in the case of the total number of antidepressants used.

**Table 2:** Correlation matrix between categories of psychotropics dissection and clinical features within 2-year of disease onset.

	**ADL**	**AUGL**	**SL**	**Number of antidepressants use**	**Number of antidepressant use (adequate dose and duration)**	**Psychiatric admission**	**Psychiatric outpatient visits**	**Psychiatric emergency visits**
ADL^#^	1							
AUGL	0.35^***^	1						
SL	0.40^***^	0.42^***^	1					
Number of antidepressants use	0.20^*^	0.21^*^	0.35^***^	1				
Number of antidepressant use (adequate dose and duration)	0.31^**^	0.25^**^	0.34^***^	0.87^***^	1			
Psychiatric admission	0.15	0.48^***^	0.11	0.19	0.20^*^	1		
Psychiatric outpatient visits	0.37^***^	0.27^**^	0.29^**^	0.37^***^	0.29^**^	0.15	1	
Psychiatric emergency visits	0.20^*^	0.15	0.24^*^	0.17	0.20^*^	0.16	0.25^*^	1

* *p < 0.05*,

** *p < 0.01*,

*** *p < 0.001*.

# *ADL, antidepressant load; AUGL, augmentation load; SL, sedative load*.

Furthermore, multiple regression analyses were conducted to examine the independent relationship between each psychotropic load and the clinical severity indicators. Before simultaneously including all psychotropic loads into the regression models, the issue of collinearity was examined by both correlation coefficients and variance inflation factor. In Table 2, the correlation coefficients rho among psychotropic loads ranged between 0.35 and 0.42. Additionally, the VIF for these 3 psychotropic loads ranged from 1.19 to 1.27. These 2 indices ensured the low risk of collinearity. Table 3 summarizes the relationship between each psychotropic loads with clinical severity indicators within the first 2 years of disease onset. When the ADL, AUGL, and SL were simultaneously included and controlling for various covariates in the models, higher ADL is related to elevated visits to the emergency and outpatient. Furthermore, the AUGL showed an independent association with the number of psychiatric admissions and outpatient visits. The higher SL showed trends of positive association with increased number of antidepressants used [IRR: 1.10, 95%CI: 1.01–1.20, *p* = 0.03], antidepressants used with ADAD [IRR: 1.11, 95%CI: 1.01–1.21, *p* = 0.02], the psychiatric emergency visits [IRR: 1.76, 95%CI: 1.09–2.85, *p* = 0.02], and outpatient visits [IRR: 1.05, 95%CI: 1.02–1.07, *p* = 0.001].

**Table 3:** Multiple regression analyses for the relationship between categories of psychotropic dissection and clinical features within 2-year of disease onset^*^.

	**Number of antidepressant use**		**Number of antidepressant use (adequate dose and duration)**		**Psychiatric emergency visits**		**Psychiatric admission**		**Psychiatric outpatient visits**	
**Defined daily dose/day**	**IRR (95%CI)**	***p***	**IRR (95%CI)**	***p***	**IRR (95%CI)**	***p***	**IRR (95%CI)**	***p***	**IRR (95%CI)**	***p***
Antidepressant load	1.06 (0.84–1.35)	0.61	1.18 (0.91–1.51)	0.21	6.20 (1.15–33.30)	0.03	1.21 (0.65–2.23)	0.55	1.24 (1.15–1.33)	<0.001
Augmentation load	0.73 (0.15–3.71)	0.71	0.72 (0.13–3.92)	0.70	0.03 (2.11E-8-37438.25)	0.62	184.76 (13.29–2568.31)	<0.001	1.68 (1.05–2.67)	0.03
Sedative load	1.10 (1.01–1.20)	0.03	1.11 (1.01–1.21)	0.02	1.76 (1.09–2.85)	0.02	0.98 (0.78–1.23)	0.88	1.05 (1.02–1.07)	0.001

*IRR, incidence rate ratio; CI, confidence interval*.

* *Covariates included into the Poisson regression model were sex, age of onset, antidepressant load, augmentation load, sedative load, comorbid anxiety disorder and number of medical diseases*.

With regards to the relationship between psychotropic loads with clinical severity indicators more than 2 years after disease onset (Table 4), the results show that the ADL independently predicted the number of psychiatric admissions [IRR (95%CI): 3.72 (1.15–12.03), *p* = 0.03], psychiatric outpatient use [IRR (95%CI): 1.25 (1.19–1.31), *p* ≤ 0.001] and severity of depressive symptoms at the end of follow-up [b (se) = 0.50 (0.19), *p* = 0.01]. The higher SL also predicted more use of psychiatric outpatient service [IRR (95%CI): 1.08 (1.06–1.10), *p* < 0.001]. In contrast, the AUGL failed to predict any clinical severity indicators beyond 2 years of disease onset.

**Table 4:** Multiple regression analyses for the relationship between categories of psychotropic dissection and clinical features beyond 2-year of disease onset.

**Psychotropic loads included in models**	Psychiatric admission^*^		Psychiatric outpatient visits^*^		**Severity of depression at the end of follow-up^#^**	
	**IRR (95%CI)**	***p***	**IRR (95%CI)**	***p***	**b (se)**	***p***
Antidepressant load	3.72 (1.15–12.03)	0.03	1.25 (1.19–1.31)	<0.001	0.50 (0.19)	0.01
Augmentation load	7.45E-5 (2.60E-11-213.91)	0.21	1.21 (0.87–1.68)	0.25	1.55 (1.29)	0.23
Sedative load	1.12 (0.74–1.68)	0.60	1.08 (1.06–1.10)	<0.001	0.03 (0.07)	0.61

*IRR, incidence rate ratio; CI, confidence interval*.

* *Covariates included into the Poisson regression model were sex, age of onset, antidepressant load, augmentation load, sedative load, comorbid anxiety disorder and number of medical diseases*.

# *Covariates included into the linear regression model were sex, age of onset, antidepressant load, augmentation load, sedative load, comorbid anxiety disorder, number of medical diseases and duration of follow-up beyond 2-year of disease onset. Severity of depression was categorized by the scores of the Chinese version of Beck Depression Inventory-II. 0–16: euthymic; 17–22: mild; 23–30: moderate and 31–63: severe*.

To examine the moderating effect of comorbid anxiety disorders on the relationship between the SL and clinical severity indicators, we included an interaction term of the SL and comorbid anxiety into all regression models. The interaction term (anxiety disorder × SL) showed statistically significant when examining the relationship between SL and the number of psychiatric outpatient visits, for both 2-year before (*p* < 0.001) and after (*p* < 0.001) the disease onset. When stratifying by the presence of anxiety disorder, the magnitude of association between SL and the outpatient services use are greater in those who were comorbid with anxiety disorders.

Because of the concern for multiple testing, a more conservative criterion for type I error was set at *p* < 0.01 to review again all significant findings regarding the SL and clinical severity indicators. With regards to the associations between SL and clinical severity indicators within 2-years of disease onset, three significant associations, including number of antidepressants used, number of antidepressants used with ADAD, and the psychiatric emergency visits did not survive under the criterion set by the Bonferroni correction. In contrast, the association between the SL and psychiatric outpatients use remained. Regarding the relationship between the SL and the clinical severity indicators beyond 2-years of disease onset, the original finding in which SL predicted higher psychiatric outpatients use, still satisfied the more conservative significance level. To illustrate the underlying mechanism that compromises the statistical significance defined by the Bonferroni-corrected criterion, additional analyses were conducted. The Supplement Tables S2, S3 illustrate how the p values changed when different sets of psychotropic loads were specified into the multiple regression models. In Supplement Table S2, when only SL was included in the model, the associations between SL and the 3 severity indicators (i.e., the number of antidepressant use, the number of antidepressant use with ADAD and psychiatric emergency visits) that became insignificant under the Bonferroni correction criterion recovered their significance. However, when adding antidepressant load, instead of augmentation load, the *p*-values for these 3 severity indicators became inflated. These findings suggest that vanished statistical significance for the 3 severity indicators with SL were mainly attributable to the confounding effects derived from the antidepressant load.

## Discussion

To the best of our knowledge, this is the first study that used pharmacological dissection and medication loads to examine the correlation between the use pattern of psychotropic agents and clinical features. It was found that the SL independently correlated with several severity indicators within 2 years of onset of major depressive disorder. Nonetheless, the higher SL only predicted more outpatients visits beyond 2 years after disease onset. These results suggest that the subgroup of MDD patients who needed intensive exposure to sedatives in the initial 2 years after the first onset of MDD may have specific pathomechanisms that cannot be resolved by antidepressants alone.

The pros and cons of using sedatives in MDD treatment remain controversial. On one hand, it is well-known that sedative use is associated with elevated risk for sedative dependency (47), emergency visits, fractures (48–50), motor vehicle crashes (51), and overdose (52). On the other hand, treatment of MDD with combined use of sedatives helps to alleviate coexisting anxiety and insomnia (20, 53), accelerate the reduction of depression symptoms (53, 54), and enhance adherence to antidepressant therapy (53). One recent large epidemiological study which used American claims data found that depressive patients who were prescribed with a long-acting sedative had a longer prescription duration and used a higher daily dosage in the initial treatment phase were more likely to become long-term sedatives users (27). This may indicate the risk of sedative dependence; however, it is also possible that it reflects the consequences of prescribing sedatives in those who needed long-term use due to unmet needs of treating depression solely by antidepressant and augmentation agents. Obviously, identifying individuals with MDD in whom the benefit of sedative use outweighs the cost would be of significant clinical relevance. We need more information to understand “who” and “why” some subgroups of patients need sedatives to control symptoms in a long-term pattern.

In the present study, the ADL, AUGL, and SL were found to positively relate to each other. Because DDD is regarded as an assumed average maintenance dose per day (33), the interlaced relationship between these three parameters suggests that if the patients with MDD required higher DDD of antidepressants to control the depressive symptoms, they would also need concomitant augmentation agents and sedatives in the treatment regimen. Undoubtedly, higher DDD for antidepressant and augmentation agents indicates more severe depressive symptoms, and the above results might suggest a subgroup of patients with more severe depression who need higher doses of sedatives. However, why they received higher SL needs further discussion.

Because sedatives are often used to mitigate the side effects of the main treatment regimen, such as anxiety and insomnia induced by monoamine antidepressants (55, 56) and akathisia induced by augmentative antipsychotics (57), a higher SL may represent a more serious side effect profile derived from the mainstay regimen. In the present study, the significant relationship of the ADL with several clinical indicators vanished when including the SL in the model specification. This suggests that the relationship between the ADL and clinical severity indicators may be either confounded or mediated by the SL. In the first instance, the higher SL dominates the associations with specific clinical severity. However, the latter instance suggests that the higher SL is secondary to the use of a higher dosage of antidepressants or augmentation agents. Under this circumstance, it would be inappropriate to specify the SL in models along with the ADL and AUGL. According to the present study's design, we could not differentiate between these two distinct patterns of “treatment difficulty” that may be encountered in the initial phase of MDD treatment.

With respect to the use of psychiatric facilities within 2 years of disease onset, the load for each class of psychotropic seemed to play different roles during the treatment course. The SL and ADL, independently from other psychotropic loads, were associated with psychiatric emergency visits, though did not survive after multiple testing correction (*p* = 0.03 and 0.02, respectively). In contrast, only a higher AUGL associated with more frequent psychiatric admissions. Psychiatric emergency visits reflect the acute and emergent domain of clinical conditions. Instead, psychiatric admissions are usually indicated when there are life-threatening conditions or there remains a suboptimal response to treatment even after a series of guideline-based intervention which could be given in outpatient services. Furthermore, the study also found that a higher SL was positively associated with the number of antidepressants with ADAD. In contrast, the ADL was not related to the number of antidepressants with ADAD in the multivariable analysis. Interestingly, these three distinct classes of psychotropic loads, which were derived from pharmacological dissection, appeared to have different clinical implications.

Although using a higher number of antidepressants with ADAD is the most common definition for treatment resistance depression (58, 59), patients with MDD may also have medication changes merely due to intolerance of adverse effects of medication, instead of poor treatment response. However, in the univariate analysis of the present study, the increasing number of antidepressants with ADAD was correlated with more use of all psychiatric facilities. This finding validates the number of antidepressants with ADAD as a robust severity indicator in the present study. Thus, our finding suggests that patients with MDD who are exposed to higher sedative loadings were more likely to have a greater level of resistance to antidepressant treatment. On the contrary, those who required a higher cumulative dosage of antidepressants (i.e., the higher ADL) to achieve adequate response were not necessarily difficult patients. These findings appear to accord with clinical experience. Furthermore, the association between the SL and number of antidepressants with ADAD also lent support against the argument that the high cumulative dosage of sedatives in MDD patients in the present study was due to the physicians' preference to use BZDs.

Because sedatives are often used to symptomatically manage anxiety disorders and comorbid anxiety disorders are a known risk factor for treatment-resistant depression (9–14), the relationship between the SL and number of antidepressants with ADAD may be confounded by comorbid anxiety disorders. Current treatment guidelines suggest that antidepressants should be the main psychotropic agents for the treatment of anxiety disorders (60, 61). In the present study, sedative use was found to correlate with several severity indicators within the first 2 years of disease onset, despite the cumulative DDD of antidepressants. This finding suggests that the high SL in MDD probably reflects that residual anxiety symptoms remained which required sedatives to make up for the inadequate response to antidepressant monotherapy. In sum, our findings suggest that a higher SL may indicate a greater level of treatment difficulty when treating first-onset MDD by antidepressants alone. Furthermore, failing to manage anxiety and insomnia symptoms with antidepressants alone and the need of using a high load of sedatives in some MDD patients also suggested that the pathomechanism of this subgroup of MDD with high SL could be hardly attributed to monoamine deficiency alone. Our findings imply that the dysfunction of the gamma-aminobutyric system may also play an important role in this specific group of patients with MDD (62, 63).

Finally, regarding predictors for clinical severity indicators more than 2 years after disease onset, the ADL replaced the SL as a more potent predictor. The ADL had a positive relationship with the frequency of psychiatric admissions, outpatient visits, and severity of depressive symptoms at the end of follow-up. This finding suggests that the AUGL and SL could only represent the algorithmic choice of augmentation agents and symptom-triggered use of medication in the initial treatment phase, respectively. Instead, the antidepressant load in the initial phase of treatment seem to foretell symptom stability in the long run. Considering the chronic and recurrent nature of MDD (64), the SL was unlikely to be a useful predictor for the long-term stability of MDD.

### Limitations

The present study had a few limitations. First, the equivalent dosages of some BZDs that have been commonly used in Taiwan are not provided in the Ashton manual. Furthermore, the estimated equivalents in the Ashton manual are derived from the clinical experience of treating BZDs' withdrawal syndrome. Such estimation is not universally accepted (34, 35, 65). Second, the DDDs for augmentation agents in the ATC are designated for their primary indications, instead of the DDD for augmentations in MDD treatment. Thus, the AUGL might not accurately reflect the magnitude of treatment that patients receive from augmentation agents. Third, the efficacy of each class of antidepressant or augmentation agent does not always have a clear dose-response relationship (66). As a result, a higher DDD-based psychotropic loading was not necessarily equal to stronger efficacy in treating MDD. Fourth, the sample size of this study is not large so that some estimates were not stable. For example, the unusual wide range of confidence interval for the association between augmentation load and clinical severity indicators. Besides, inadequate statistical power, which is also related to sample size, contributed to the failure of several significant findings to survive under a more conservative criterion of type I error. Enlarging the sample size is mandatory in the future. On the contrary, because this study intended to explore the relationship between psychotropics loads and different features of clinical severity indicators, multiple tests are inevitable and should be informative with regards to the scientific merit. In fact, the Bonferroni procedure reduces power and increasing type II error to unacceptable levels. It has been suggested that the effect size along with confidence interval, as provided in the present study, are more useful than the *p*-value (67). Finally, the study cohort only included patients with at least 2 years of complete treatment course. Those patients who had more severe depressive symptoms, poor medication adherence, or doctor-shopping behaviors may not be included. Thus, the representativeness of participants in the present study may be compromised and our findings should be generalized cautiously.

## Conclusion

By dissecting the psychotropic agents that had been used in the initial treatment phase of first-onset MDD, the present study illustrates the independent and specific association between the high loading of sedatives and clinical severity indicators of MDD. This finding suggests that the SL is a potential phenotypic specifier that could serve to subgroup patients with MDD. In the future, further endophenotypic and genotypic studies which apply the SL-based phenotypes are necessary to examine the biological validity of this novel specifier.

## Data Availability

The raw data supporting the conclusions of this manuscript will be made available by the authors, without undue reservation, to any qualified researcher.

## Ethics Statement

This study was approved by the research ethics committees of National Taiwan University Hospital, Taipei Municipal Wan-Fang Hospital, and Taipei City Hospital, Songde Branch. Written informed consent was obtained from each participant.

## Author Contributions

All the authors are members of RECOGNIZE-TRD Program. Y-CW, H-TL, and H-CC drafted the manuscript. P-HK and H-CC conceived and designed the study, and critically revised the manuscript. H-CC owns primary responsibility for the final content. M-LL, M-CH, and C-HC assisted to refer the patients as participants. M-LL, M-CH, C-HC, T-HW, SW, and W-CM gave critical opinions on the study design and the manuscript. All authors approved the final manuscript.

### Conflict of Interest Statement

The authors declare that the research was conducted in the absence of any commercial or financial relationships that could be construed as a potential conflict of interest.
