# Supplementary material for: The Association Between the Sedative Loads and Clinical Severity Indicators in the First-Onset Major Depressive Disorder
Source: Front Psychiatry. 2019 Mar 18;10:129. doi: 10.3389/fpsyt.2019.00129 (PMC6431631; doi:10.3389/fpsyt.2019.00129)
Supplement: Supplementary file 1 [file Data_Sheet_1.docx]

The Association between the Sedative Loads and Clinical Severity Indicators in the First-onset Major Depressive Disorder

| **Supplement table S1. The equivalent oral dosage of sedatives** | |
| --- | --- |
|  | Equivalent oral dosage |
| **Benzodiazepines** |  |
| Alprazolam | 0.5 |
| Bromazepam | 6 |
| Brotizolam | 0.5 |
| Chlordiazepoxide | 25 |
| Clobazam | 20 |
| Clonazepam | 0.5 |
| Clorazepate | 15 |
| Diazepam | 10 |
| Estazolam | 1 |
| Fludiazepam | 0.5 |
| Flunitrazepam | 1 |
| Flurazepam | 15 |
| Halazepam | 20 |
| Lorazepam | 1 |
| Lormetazepam | 2 |
| Medazepam | 10 |
| Midazolam | 5 |
| Nitrazepam | 10 |
| Nordazepam | 10 |
| Oxazepam | 20 |
| Oxazolam | 20 |
| Quazepam | 20 |
| Temazepam | 20 |
| Triazolam | 0.5 |
| **Non-benzodiazepines** |  |
| Zaleplon | 20 |
| Zolpidem | 20 |
| Zopiclone | 15 |
| Eszopiclone | 3 |

| **Supplement table S2. Multiple regression analyses for the relationship between categories of psychotropic dissection and clinical features within 2-year of disease onset *** | | | | | | | | | | |
| --- | --- | --- | --- | --- | --- | --- | --- | --- | --- | --- |
| Psychotropic loads included in models  (Defined daily dose/ day) | Number of  antidepressant use | | Number of antidepressant use  (adequate dose and duration) | | Psychiatric  emergency visits | | Psychiatric  admission | | Psychiatric  outpatient visits | |
|  | IRR  (95%CI) | *p* | IRR  (95%CI) | *p* | IRR  (95%CI) | *p* | IRR  (95%CI) | *p* | IRR  (95%CI) | *p* |
| **Individual loads** |  |  |  |  |  |  |  |  |  |  |
| Antidepressant load | 1.17  (0.94-1.46) | 0.15 | 1.31  (1.04-1.64) | 0.02 | 12.18  (2.44-60.9) | 0.002 | 1.43  (0.82-2.50) | 0.21 | 1.32  (1.23-1.41) | <0.001 |
| Augmentation load | 1.51  (0.34-6.59) | 0.59 | 1.78  (0.39-8.20) | 0.50 | 39.4  (0.04-35741.61) | 0.29 | 192.27  (17.13-2158.52) | <0.001 | 2.94  (1.93-4.49) | <0.001 |
| Sedative load | 1.10  (1.02-1.19) | 0.01 | 1.13  (1.04-1.22) | 0.003 | 1.85  (1.30-2.63) | 0.001 | 1.16  (0.96-1.40) | 0.14 | 1.09  (1.06-1.11) | <0.001 |
| **Two loads** |  |  |  |  |  |  |  |  |  |  |
| Antidepressant load | 1.06  (0.83-1.35) | 0.63 | 1.17  (0.91-1.50) | 0.22 | 6.29  (1.14-34.64) | 0.04 | 1.27  (0.69-2.33) | 0.45 | 1.25  (1.16-1.34) | <0.001 |
| Sedative load | 1.09  (1.01-1.19) | 0.03 | 1.10  (1.01-1.20) | 0.03 | 1.64  (1.12-2.41) | 0.01 | 1.12  (0.91-1.38) | 0.29 | 1.05  (1.03-1.08) | <0.001 |
|  |  |  |  |  |  |  |  |  |  |  |
| Augmentation load | 0.76  (0.15-3.81) | 0.74 | 0.78  (0.15-4.19) | 0.77 | 0.04  (8.39E-8-21539.70) | 0.64 | 187.40  (13.78-2548.96) | <0.001 | 1.88  (1.18-2.97) | 0.01 |
| Sedative load | 1.11  (1.02-1.20) | 0.01 | 1.13  (1.04-1.23) | 0.004 | 1.95  (1.26-3.03) | 0.003 | 1.01  (0.81-1.24) | 0.96 | 1.07  (1.05-1.10) | <0.001 |
| Abbreviations: IRR: incidence rate ratio; CI: confidence interval | | | | | | | | | | |
| *: Covariates included into the Poisson regression model were sex, age of onset, comorbid anxiety disorder and number of medical diseases | | | | | | | | | | |

| **Supplement table S3. Multiple regression analyses for the relationship between categories of psychotropic dissection and clinical features beyond 2-year of disease onset *** | | | | | | |
| --- | --- | --- | --- | --- | --- | --- |
| Psychotropic loads included in models | Psychiatric  admission* | | Psychiatric  outpatient visits* | | Severity of depression at the end of follow-up^#^ | |
|  | IRR  (95%CI) | *p* | IRR  (95%CI) | *p* | b (se) | *p* |
| **Individual loads** |  |  |  |  |  |  |
| Antidepressant load | 4.22  (1.26-14.15) | 0.02 | 1.33  (1.27-1.39) | <0.001 | 0.58  (0.18) | 0.001 |
| Augmentation load | 0.003  (2.30E-8-374.95) | 0.33 | 1.84  (1.36-2.49) | <0.001 | 2.52  (1.25) | 0.05 |
| Sedative load | 1.32  (0.92-1.88) | 0.13 | 1.12  (1.09-1.14) | <0.001 | 0.13  (0.06) | 0.04 |
| **Two loads** |  |  |  |  |  |  |
| Antidepressant load | 3.87  (1.12-13.30) | 0.03 | 1.25  (1.19-1.31) | <0.001 | 0.52  (0.19) | 0.01 |
| Sedative load | 1.11  (0.78-1.59) | 0.56 | 1.08  (1.06-1.10) | <0.001 | 0.06  (0.06) | 0.37 |
|  |  |  |  |  |  |  |
| Augmentation load | 0.001  (3.10E-9-69.90) | 0.21 | 1.16  (0.84-1.60) | 0.37 | 1.85  (1.33) | 0.17 |
| Sedative load | 1.48  (1.00-2.09) | 0.05 | 1.11  (1.09-1.14) | <0.001 | 0.10  (0.07) | 0.15 |
| Abbreviations: IRR: incidence rate ratio; CI: confidence interval | | | | | | |
| *: Covariates included into the Poisson regression model were sex, age of onset, comorbid anxiety disorder and number of medical diseases | | | | | | |
| ^#^: Covariates included into the linear regression model were sex, age of onset, comorbid anxiety disorder, number of medical diseases and duration of follow-up beyond 2-year of disease onset. Severity of depression was categorized by the scores of the Chinese version of Beck Depression Inventory-II. 0-16: euthymic; 17-22: mild; 23-30: moderate and 31-63: severe | | | | | | |
