# Supplementary material for: The Association Between the Sedative Loads and Clinical Severity Indicators in the First-Onset Major Depressive Disorder
Source: Front Psychiatry. 2019 Mar 18;10:129. doi: 10.3389/fpsyt.2019.00129 (PMC6431631; doi:10.3389/fpsyt.2019.00129)
Supplement: Supplementary file 2 [file Image_1.pdf]

Supplement Figure S1. Timetable of recording procedure during study and follow-up period

Time after being diagnosed as first-onset depression	1st month	2nd month	3rd month	4-24 months	after 24 months	
					Being invited to this study	End of follow-up
**The medication being prescribed**	Antidepressant A<br>BZD A	Antidepressant A<br>Augmentation A<br>BZD A	Antidepressant A<br>Antidepressant B<br>Augmentation A<br>BZD A	…<br>…<br>…<br>…		
**Pharmacological dissection**						
Antidepressant load	DDD of antidepressant A	DDD of antidepressant A	DDD of antidepressant A+antidepressant B	…		
Augmentation load		DDD of augmentation A	DDD of augmentation A	…		
Sedative load	Equivalent dose of BZD A	Equivalent dose of BZD A	Equivalent dose of BZD A	…		
**Clinical indicator**						
Number of antidepressant use<br>Emergency visits<br>Admission<br>OPD visits	Being recorded by chart review				Keeping follow-up	
Severity of depression						Evaluated by BDI score
